# A Comparative Study between Olanzapine and Risperidone Regarding Drug-Induced Electrocardiographic Changes

**DOI:** 10.1155/2014/637016

**Published:** 2014-09-09

**Authors:** Saeed Shoja Shafti, Parisa Fallah Jahromi

**Affiliations:** ^1^University of Social Welfare and Rehabilitation Sciences (USWR), Razi Psychiatric Hospital, P.O. Box 18735-569, Tehran 18664 24336, Iran; ^2^Internal Ward, Razi Psychiatric Hospital, Tehran 18664 24336, Iran

## Abstract

*Introduction*. Among atypical antipsychotics, none has been linked to torsade de pointes. In the present study, the electrocardiographic changes induced by olanzapine have been compared with risperidone. *Method and Materials*. 268 patients were entered into an open study for random assignment to olanzapine or risperidone. ECG was taken at baseline and at the end of the treatment. The parameters that had been assessed included Q-T interval (corrected = Q-Tc) and other related parameters. Correction of the observed Q-T interval was done according to Frederica's formula (QTcF). *Results*. While 14.86% and 25% of the cases in the olanzapine group showed prolongation and shortening of QTcF, respectively, comparable changes in the risperidone group were restricted to its prolongation (32.5%). Comparison of means between baseline QTcF of risperidone group versus its posttreatment measurement showed a significant increment (*P* = 0.02). Also, the quantity of cases with shortening of QTcF in the olanzapine group was significantly more than its opposite (*P* = 0.02). *Conclusion*. Comparable propensity of olanzapine and risperidone for induction of electrocardiographic changes demands adequate cautiousness by clinicians, particularly with respect to shortening of Q-T interval, which was mainly noticeable in the olanzapine group.

## 1. Introduction

A study comparing the risk of sudden cardiac death of FGAs and SGAs found that patients taking any antipsychotic medication have at least a twofold greater risk for sudden cardiac death compared to patients who are not treated with these medications [1]. Although multiple factors may contribute to this increased risk, perhaps the most reasonable explanation is that antipsychotic medications cause an increase of serious ventricular arrhythmias, likely via potassium channel blockade and prolonged cardiac repolarization [1]. While the relationship between QTc prolongation, sudden cardiac death, arrhythmias, and antipsychotic medications need to be elucidated; these medications do have the potential to prolong the QT interval to varying levels. In a study thioridazine and ziprasidone were associated with the greatest increases in QTc from baseline using Bazett's formula, while quetiapine, risperidone, olanzapine, and haloperidol caused smaller increases in QTc [2]. Studies have shown that QTc intervals of over 440 ms and especially over 500 msec are associated with an increased risk of sudden death [3, 4]. A number of antipsychotics have been linked to torsade de pointes and sudden death, among them pimozide, sertindole, droperidol, haloperidol, and thioridazine [5]. In a large retrospective case-control study of all sudden deaths in psychiatric wards of five hospitals in England over 11 years, the only antipsychotic that was found to be an independent risk factor for sudden death was thioridazine [6]. Of six major antipsychotics (thioridazine, ziprasidone, quetiapine, risperidone, olanzapine, and haloperidol), thioridazine produces the largest QTc prolongation, with a mean change of 35.6 ms [7, 8]. Among the atypical antipsychotics, none has been linked to torsade de pointes, even though most of them have a larger impact on QTc than haloperidol, which has been linked to torsade de pointes. Risperidone has been linked to one fatality due to pulseless electrical activity, and it has been reported to cause QTc and QRS prolongation in two cases of overdose and in 8 of 380 patients in a double-blind study by the manufacturer [9]. The fatality may have been due to factors other than QTc prolongation [10], since for example, mental arithmetic as well may induce prolongation of QT interval and other ECG-derived indices of arrhythmic vulnerability [11]. So far, aripiprazole, olanzapine, and quetiapine have not been linked to torsade de pointes. The upper limits of normal QTc interval, corrected for heart rate by Bazett's formula (QTcB) and recommended in the first regulatory guidance, were 450 ms for adult men and 470 ms for adult women (Moss, 1993; CPMP, 1997). According to Drew et al. [12], a normal QTc interval is <450 ms in men and <460 ms in women. The report from ACC/AHA/HRS on “Key Data Elements and Definitions for Electrophysiological Studies and Procedures” defines the upper limit of QTc interval at 440 ms for adult men and 460 ms for adult women [13]. From a large dataset of ECGs of 46 129 individuals with a very low probability of cardiovascular disease and using the 2nd and 98th percentiles, Mason et al. [14] have determined normal reference range to be 361–457 ms for QTcB interval and 359–445 ms for Fridericia-corrected QTc (QTcF) interval [15]. In the present study, electrocardiographic changes induced by olanzapine and risperidone have been compared with each other.

## 2. Method and Materials

Two hundred and sixty eight female inpatients with diagnosis of schizophrenia, according to the criteria of* Diagnostic and Statistical Manual of Mental Disorders*, 4th edition, Text Revision, were entered in either of the two parallel groups, to participate in an open study for random assignment to olanzapine (*n* = 148, 5–25 mg/day) or risperidone (*n* = 120, 4–8 mg/day). After complete description of the study to the subjects, written informed consent was obtained from either the participant or a legal guardian or representative. Also, the patients were free to stop the medication if they wished. In addition, the whole procedure was approved by the related ethical committee of the university. Based on primary clinical and laboratory checkups, any patient with any diagnosed medical (like severe renal or liver disease) or cardiovascular problem (like tachycardia, bradycardia, interventricular conduction defect, ischemic heart disease, myocardial or pericardial disease, congestive heart failure), electrolyte disturbances (hypocalcaemia, hypercalcemia, hypomagnesaemia, hypokalemia, and hyperkalemia), thyroid dysfunction, cerebral or subarachnoid injury, and also patients who were utilizing other concomitant drugs (like digitalis that shortens Q-Tc or quinidine, procainamide, and amiodarone that increase that, or utilization of mood stabilizers, antidepressants, or depot antipsychotics) or cases more than 40 years old had been excluded from the trial. Thus, the aim of the study was to determine electrocardiographic changes in healthy schizophrenic patients, who were previously on a variety of typical antipsychotics. So, after a minimum 7-day washout period, both of these drugs were prescribed according to practice guidelines and standard-titration protocols [16] and in accordance to the following regimen: 1 mg/day of risperidone or 5 mg/day of olanzapine at baseline up to 2 mg/day of risperidone and 10 mg/day olanzapine at the end of the first week. Weekly interval increments of 2 mg for risperidone and 5 mg for olanzapine, individually and according to clinical situation, up to maximum of 8 mg and 25 mg for risperidone and olanzapine, respectively at week 5. The 5th week dosage remained constant up to the end of the study. Standard 12-lead surface ECG was taken from each patient at baseline, before initiation of the antipsychotic, and then again at the end of the treatment, just before discharge (in the sunrise, before initiation of daily prescription). No other psychotropic drug was permitted during the assessment. The parameters that had been assessed included heart rate (HR), P-R interval, QRS interval, Q-T interval (corrected = Q-Tc), ventricular activation time (VAT), ST segment, T wave, axis of QRS, and finally interventricular conduction process. Although it is a standard practice to measure the QT interval from the beginning of the QRS complex to the end of the T wave, but the actual methods of measurement have not been standardized yet and in addition dissimilar opinions exist regarding the most useful method for correction of Q-T interval for heart rate (such as* Bazett formula*,* Fridericia cube-root correction,* or* Framingham linear regression equation*) [17]. In this experiment, measurement of Q-T interval was based on* “expert opinion guidelines for measuring the QT interval”* [17]. Also, calculation of heart rate was based on the* “modified table of Ashman R and Hull E,”* and correction of the observed Q-T interval was done according to Frederica's formula (QTcF = using the cube-root of RR) for rate correction of Q-T interval [18]. Since the purpose of the current assessment was to detect drug induced changes of ECG, so no definite criterion was set for a clinically meaningful alteration in Q-Tc or other related parameters.

## 3. Statistical Analysis

Patients were compared on baseline characteristics by “chi-square test” for categorical variables and “*t*-test” for continues variables. Also, the results were analyzed by “*t*-test” or “compression of two proportions” for intragroup and between-group analysis. Statistical significance was defined as a 2-sided *P* value ≤ 0.05. MedCalc version 9.4.1.0 and OpenStat version 1.0.0.0 were used as statistical software tools for analysis.

## 4. Results

Groups were initially comparable and demographic and diagnostic variables were analogous (Table 1). 39.86% (*n* = 59) of the cases in the olanzapine group and 32.5% (*n* = 39) of them in the risperidone group showed some Q-T interval (QTcF) changes (comparing baseline to post-treatment stage). In addition, 14.86% (*n* = 22) and 26.35% (*n* = 39) of the patients in the olanzapine group showed prolongation (0.01–0.04 Sec, mean = 0.02 ± 0.01 Sec) and shortening (0.01–0.04 Sec, mean = 0.02 ± 0.01 Sec) of QTcF, respectively. This decrease in QTcF was equal to 0.04 Sec in at least 6.08% (*n* = 9) of the patients. The abovementioned changes in the risperidone group was restricted to only prolongation of QTcF (0.01-0.02 Sec, mean = 0.016 ± 0.005 Sec). Comparison of proportions, between olanzapione and risperidone, regarding total number of cases in the related groups with QTcF changes, was nonsignificant (*z* = 1.24, *P* = 0.21, 95% CI = −0.04, 0.18). Besides, in the olanzapine group, comparison of means between baseline QTcF versus its posttreatment measurement and also posttreatment QTcF in the olanzapine group against comparable variable in the risperidone group were nonsignificant. But comparison of means, between baseline QTcF of risperidone group versus its posttreatment measurement showed a significant increment (*P* = 0.002) (Table 2). In addition, comparison of proportions in the olanzapine group showed that the quantity of cases with shortening of QTcF was significantly more than the number of the patients with QTcF prolongation (*z* = −2.44, *P* = 0.01, 95% CI = −0.20, −0.02). Moreover, 5.40% (*n* = 8) of the patients in the olanzapine group showed alteration of P-R interval. Four of them showed prolongation (0.02 Sec) and the other ones shortening of that (0.02 Sec). But at the end, such an alteration was nonsignificant, in comparison with baseline, in the related group (*P* = 0.15) (Table 2). In addition, in the later group, two of them had synchronized increment of QTcF and P-R interval. It is mentionable that there was no P-R alteration in the risperidone group. Intragroup analysis did not show any significant difference in HR, VAT, and QRS complex between baseline and final stage of the treatment, in both of the aforesaid groups (Table 2). Moreover, no shifting in the S-T segment (depression or elevation) or T wave's alteration was evident among those cases. In the olanzapine group, two patients showed left anterior hemiblock, in accompany with mild shortening of QTcF (0.01 Sec). No serious adverse effect, like torsade de pointes, Brugada syndrome, ventricular tachyarrhythmia, ventricular fibrillation, and sudden death, occurred throughout this trial. The mean modal dose of olanzapine during the present assessment was 19.49 ± 5.51 mg/day. The most common dosages of olanzapine were 20 mg/day (*n* = 98, 66.21%), 25 mg/day (*n* = 26, 17.56%), and 15 mg/day (*n* = 24, 16.21%). The mean modal dose of risperidone throughout the experiment was 5.14 ± 2.86. Its most common doses were 6 mg/day (*n* = 58, 48.33%), 8 mg/day (*n* = 48, 40%), and 4 mg/day (*n* = 16, 13.33%). Also during the study, 26.66% (*N* = 32) of the cases in the risperidone group and 9.45% (*N* = 14) of them in the olanzapine group showed extrapyramidal side effects. Increase in weight was significantly greater in the patients treated with olanzapine (*n* = 34, 22.97%) than in those treated with risperidone (*n* = 10, 8.33%). The mean weight gain by olanzapine and risperidone was about 2.2 ± 0.91 kg and 0.6 ± 0.75 kg, respectively. Post hoc power analysis demonstrated an acceptable power of 0.88 for this trial.

## 5. Discussion

According to the findings, in posttreatment ECG of patients, more different changes were obvious in the olanzapine group, in comparison with the risperidone group. These alterations included mostly shortening or prolongation of QTcF, as well as left anterior hemiblock and P-R interval shortening or prolongation. Moreover in the olanzapine group, there was significantly more number of cases with QTcF shortening, in comparison with its prolongation. But conversely, significant increment of mean QTcF had been induced only by risperidone. So, while our results were more or less in harmony with the findings of Ravin and Levenson [9] and Yerrabolu et al. [19] as regards the effect of risperidone on Q-T interval, it is against Czekalla et al. [20] who stated that “risperidone can be used safely in elderly patients, who are often taking several medications, without risk of increased Q-T dispersion.” Also, with respect to olanzapine, while our results were not in agreement with Janion et al., who stated that “olanzapine is relatively safe and does not contribute significantly to a QTc prolongation that could result in potentially fatal ventricular arrhythmias” [21], it is relatively in accord with Mehul Desai et al., regarding potentiality of olanzapine for induction of Q-T interval prolongation and ventricular fibrillation [22].

In essence, in considering the use of drugs that may prolong the QT interval, factors to be reviewed include a family or personal history of long QT syndrome, a history of sudden cardiac arrest, syncope or unexplained seizure, arrhythmias, hypertension, valvular heart disease, bradycardia, and use of other medications that may prolong the QT interval or interfere with the metabolism of QT-prolonging agents [23]. On the other hand, shortening of QTc by olanzapine had not been expressed before in the literature. Basically, little information is known about the issue of drug-induced QT/QTc shortening [24]. For example, Algra et al. (1993) studied the effects of variability in the duration of the QTc interval on the occurrence of sudden death in a nested case-control study. They reported that patients with a prolonged mean QTc interval of >440 ms over 24 h had a 2.3-fold higher risk of dying suddenly than patients with a normal mean QTc (400–440 ms). Perhaps a little surprising at the time was the finding that patients with a shortened mean QTc (<400 ms) also had a higher risk (relative risk 2.4) compared with those who had a mean QTc values in the range 400–440 ms [25]. Likewise, Viskin et al. (2004) have reported from a case-control study that, despite a significant overlap, the QTc interval of male patients with idiopathic VF was shorter than the QTc interval of otherwise healthy male people (371 ± 22 ms versus 385 ± 19 ms, *P* = 0.034) [26].

As with QT/QTc prolongation, there are genetic syndromes and pharmaceutical agents which may cause shortening of QT/QTc. Although the potential safety issue of QT/QTc shortening and its suitability as a biomarker of drug-induced cardiac arrhythmias are indistinguishable, however, the type of arrhythmia associated with prolongation and shortening seems to differ. Prolongation is associated with TdP, whereas shortening of QT/QTc is proposed to be associated mainly with ventricular fibrillation (VF). Current clinical epidemiological evidence suggests that excessive shortening of QT/QTc may facilitate induction of VF [24]. Acquired QT/QTc shortening has been reported to be caused by hypercalcemia, hyperkalemia, hyperthermia, and myocardial ischemia, which totally had been ruled out by initial checkups in the present assessment. But nevertheless, since the sample included only healthy female schizophrenic patients, then there is limitation against generalization of its findings. Also, normal variation in cardiac parameters within individuals must be taken into view, because even Q-Tc can vary considerably in 2 ECGs gathered minutes apart. But anyhow, if cardiac safety of drugs is a matter of concern, then the nonclinical observations on drug-induced shortening of action potential duration (APD) and/or QT interval and the associated profibrillatory effects discussed earlier should be a matter of concern and should be explored further rather than underestimating their potential significance. There is a very obvious role for investigating the effect of QT-shortening drugs on transmural dispersion of repolarization, triangulation of action potential, and beat-to-beat variability in repolarization when determining their proarrythmic potential. In addition, it is mentionable that, as with diagnosing congenital SQTS, Bazett's formula seems inappropriate for making a diagnosis of drug-induced QT interval shortening, since its estimation depends directly on baseline values [15, 27]. Short duration of study (limited to the period of acute treatment), gender-based sampling, lack of placebo arm, which may have significant impact on the assay sensitivity of the study, and a bit small sample size were among the prominent weaknesses of this trial. Further analogous trials in future can improve our knowledge with respect to this vital subject.

## 6. Conclusion

Comparable propensity of olanzapine and risperidone for induction of electrocardiographic changes demands adequate cautiousness by clinicians, particularly with respect to shortening of Q-T interval that was mainly noticeable in the olanzapine group.

## Figures and Tables

**Table 1:** Baseline demographic and electrocardiographic characteristics of the participants.

Variables	Olanzapine (*n* = 148)	Risperidone (*n* = 120)	*X*2	*t*	df	*P*	95% CI
Gender, female	100%	100%					
Age, mean of years	25.63 ± 6.01	23.92 ± 5.87		1.74	266	0.08	−3.64, 0.22
Married cases	118	94	0.279		1	0.77	−0.08, 0.11
Duration of treatment prior to discharge, mean of days	24.54 ± 22	20.7 ± 7.25		1.39	266	0.16	−9.286, 1.60
Baseline HR	86.91 ± 27.5	92 ± 21.5		1.24	266	0.21	−2.99, 13.17
Baseline P-R interval	0.13 ± 0.06	0.14 ± 0.05		1.09	266	0.27	−0.008, 0.02
Baseline QTcF	0.40 ± 0.04	0.40 ± 0.02		0.00	266	1.00	−0.01, 0.01
Baseline QRS complex	0.08 ± 0.02	0.07 ± 0.02		1.82	266	0.07	−0.012, 0.0005
Baseline VAT	0.03 ± 0.01	0.02 ± 0.007		1.39	266	0.16	−0.004, 0.0008

HR: heart rate, QTcF: corrected QT by Fridericia's formula, and VAT: ventricular activation time.

**Table 2:** Intragroup analysis of different parameters between baseline and final stage of the assessment.

Drug∖variable	Mean or number at baseline	Mean or number at ending	*t*	df	*P*	CI
Olanzapine-HR	86.91 ± 27.5	84.45 ± 22.5	0.84	294	0.40	−3.29, 8.21
Risperidone-HR	92 ± 21.5	89 ± 21	1.09	238	0.27	−2.40, 8.40
Olanzapine-P-R interval	0.13 ± 0.06	0.14 ± 0.06	−1.4	294	0.15	−0.02, 0.00
Risperidone-P-R interval	0.14 ± 0.05	0.14 ± 0.05			>0.05	
Olanzapine-QRS	0.08 ± 0.02	0.082 ± 0.02			>0.05	
Risperidone-QRS	0.07 ± 0.02	0.079 ± 0.02			>0.05	
Olanzapine-VAT	0.03 ± 0.01	0.030 ± 0.01			>0.05	
Risperidone-VAT	0.02 ± 0.007	0.028 ± 0.007			>0.05	
Olanzapine-QTcF	0.40 ± 0.04	0.41 ± 0.06	−1.68	294	0.09	−0.02, 0.00
Risperidone-QTcF	0.40 ± 0.02	0.41 ± 0.03	−3.03	238	0.002	−0.02, −0.00
Olanzapine-Normal axis (Vector)	100% (*n* = 148)	97.29% (*n* = 144)			0.44	
Risperidone-Normal axis (Vector)	100% (*n* = 120)	100% (*n* = 120)			>0.05	
Olanzapine-normal interventricular conduction	100% (*n* = 148)	97.29% (*n* = 144)			0.44	
Risperidone-normal interventricular conduction	100% (*n* = 120)	100% (*n* = 120)			>0.05	
Olanzapine- upright T wave	100% (*n* = 148)	100% (*n* = 148)			>0.05	
Risperidone-upright T wave	100% (*n* = 120)	100% (*n* = 120)			>0.05	

HR: heart rate, QTcF: corrected QT by Fridericia's formula, and VAT: ventricular activation time.
